# The Fatty Acid Profile in Patients with Newly Diagnosed Diabetes: Why It Could Be Unsuspected

**DOI:** 10.1155/2017/6424186

**Published:** 2017-09-11

**Authors:** C. Castro-Correia, S. Sousa, S. Norberto, C. Matos, V. F. Domingues, M. Fontoura, C. Calhau

**Affiliations:** ^1^Unidade de Endocrinologia Pediátrica, Hospital Pediátrico Integrado, Centro Hospitalar S. João, Faculty of Medicine, University of Porto, 4200 Porto, Portugal; ^2^REQUIMTE/LAQV-GRAQ, Instituto Superior de Engenharia do Porto, Instituto Politécnico do Porto, 4200-072 Porto, Portugal; ^3^Department of Biochemistry, Faculty of Medicine, University of Porto, Centro de Investigação Médica, 4200-450 Porto, Portugal; ^4^Center for Research in Health Technologies and Information Systems (CINTESIS), 4200-450 Porto, Portugal

## Abstract

**Context:**

Several studies have shown a link between proinflammatory activity and the presence or deficit of some fatty acids. Inflammation is associated with several diseases including diabetes.

**Objective:**

To characterize and compare the fatty acids profiles in children with inaugural type 1 diabetes, diabetic children (at least 1 year after diagnosis), and healthy children.

**Design:**

Plasma fatty acids profiles in children with inaugural diabetes, children with noninaugural diabetes, and controls, all of whom were prepubescent with a BMI < 85th percentile, were evaluated.

**Results:**

Omega-3 fatty acid levels were higher in recently diagnosed subjects with diabetes than in controls. The ratio of omega-6/omega-3 fatty acids was higher in the control population. Omega-6 fatty acid levels were higher in the nonrecent diabetic subjects than in the children with recently diagnosed diabetes, and the levels were higher in the nonrecent diabetes group compared to the control group.

**Conclusion:**

Our findings showed higher levels of alpha-linolenic acid, EPA, and DHA, as well as mono- and polyunsaturated fatty acids, in diabetic children. These findings reinforce the importance of precocious nutritional attention and intervention in the treatment of diabetic children.

## 1. Introduction

Type 1 diabetes is an autoimmune disease triggered by the destruction of pancreatic beta cells. It is usually preceded by the emergence of autoimmunity, including anti-GAD antibodies (glutamine decarboxylase). Both genetic and environmental factors contribute to the development of the disease [1].

Several studies have demonstrated a link between proinflammatory activity and the presence or relative deficit of some fatty acids [2, 3]. Thus, a low level of omega-3 fatty acids, which occurs frequently in western diets, appears to promote an inflammatory response. In fact, higher intake of omega-3 fatty acids and a high concentration of these fatty acids in the erythrocyte membrane are associated with a lower risk of developing pancreatic beta-autoimmunity [4].

It is also known that there is a relationship between inflammatory activity and the presence of type 1 diabetes-associated complications such as retinopathy or diabetic nephropathy. A diet rich in omega-3 fatty acids appears to be beneficial through the promotion of greater insulin sensitivity and improved glucose metabolism [5]. These fatty acids decrease inflammation by altering the transcription of genes involved in the inflammatory response (e.g., NF-kB, INF-*γ*) and by competing with the binding of omega-6 fatty acids to enzymes involved in the synthesis of proinflammatory eicosanoids [6]. Eicosanoids are biologically active lipid mediators that regulate inflammation [7] and include prostaglandins, prostacyclins, thromboxanes, lipoxins, and leukotrienes [8]. The presence of high levels of prostaglandin E2 appears to promote the loss of beta cells and inhibits beta cell proliferation. Prostaglandin E2 also reduces insulin secretion and insulin sensitivity through its binding to the EP3 receptor [9].

*α*-Linolenic acid is the primary omega-3 fatty acid present in western diets and is found especially in vegetables, namely, in soy [1]. Eicosapentaenoic acid (EPA) and docosahexaenoic acid (DHA) are found in fat fish that is frequently consumed in the Mediterranean diet.

Omega-6 fatty acids promote inflammation. The main acid of this type in the western diet is linoleic acid, which is present mainly in vegetable oils, and arachidonic acid, which is derived from meat.

While the clinical follow-up of children and young people with type 1 diabetes involves the periodic evaluation of their lipid profile, namely, total cholesterol, HDL, LDL, and triglycerides, the concentrations of different types of fatty acids have not been investigated.

To address this gap in knowledge, the authors aimed to study the differences between the fatty acid profiles in children with inaugural type 1 diabetes, diabetic children (at least 1 year after diagnosis), and healthy children.

## 2. Methods

### 2.1. Human Sample

Between 2012 and 2013, we obtained plasma fatty acids profiles from prepubescent children with inaugural diabetes and with a BMI < 85th percentile at the time of their diagnosis (*N* = 8). Ages ranged from 4.8 to 9.6 years (average: 8.47). We also included diabetic children who attended the Pediatric Endocrinology Unit of S. João Hospital (*N* = 34) who were between 2.8 and 10.4 years of age (average: 7.06). Children were excluded if they had a BMI above the 85th percentile, were diagnosed less than six months prior to enrolment, had other associated diseases, or were taking any other medication. Children with a Tanner stage ≥ 2 were also excluded.

The control population included children who were scheduled to undergo planned surgical interventions, had no chronic diseases, and were not taking any medication. Children with a BMI > 85th percentile or who were pubescent were also excluded. These children were between 3.5 and 8.6 years of age (average: 6.1) (Table 1).

The study was approved by the hospital's ethics committee. Authorization and informed consent were obtained from the parents of all the children participating in the study.

### 2.2. Chemicals and Reagents

Sodium hydroxide and anhydrous sodium sulfate were obtained from Pronalab® (Lisbon, Portugal); boron trifluoride in methanol (14% in methanol) and butylated hydroxytoluene (BHT) (≥99%) were from Sigma-Aldrich® (St. Louis, USA); *n*-hexane (99%) was from Merck® (Darmstadt, Germany); methanol was from VWR Chemicals Prolabo® (Fontenay-sous-Bois, France); and sodium chloride (99.5%) was from Panreac® (Barcelona, Spain).

### 2.3. Determination of Plasma Fatty Acids Profiles by Gas Chromatography Coupled to a Flame Ionization Detector

To analyze the plasma fatty acids profiles, the boiling point must first be lowered which is achieved by the derivatization of the fatty acids [10]. In this case, sodium methoxide (NaOMe) is used as a catalyst to form fatty acid methyl esters (FAMEs) [11] which have a lower boiling point.

The plasma samples were placed in tubes with 20 *μ*g of internal standard (C13:0), and then 5 mL of NaOMe (0.5 M) was added, and the tube was vigorously shaken. The samples were heated to 100°C for 10 min and cooled for 5 min on ice. After cooling, 5 mL of boron trifluoride-methanol was added to the samples, which were again heated to 100°C for 30 min and cooled for 5 min on ice. Then, 600 *μ*L of *n*-hexane with butylated hydroxytoluene (BHT) (0.02%) was added to prevent lipid oxidation. The tubes were vortexed, and 2 mL of sodium chloride was added; then, the tubes were centrifuged for 10 min at 2200 rpm. The top layer was retrieved and dried with anhydrous sodium sulfate. Then, 100 *μ*L was removed and evaporated to dryness with nitrogen and finally rediluted in 75 *μ*L of *n*-hexane [12].

Gas chromatography analyses were performed using a Shimadzu GC-2010 equipped with a flame ionization detector and a Shimadzu AOC-20i autoinjector. The separation of FAMEs was carried out on an Agilent® J&W Cp-Sil 88 capillary column (50 m × 0.25 mm ID, 0.20 *μ*m) from Santa Clara, USA. The operating conditions were as follows: the split–splitless injector was used in split mode with a split ratio of 1 : 50. The injection volume of the sample was 1.5 *μ*L. The injector and detector temperatures were kept at 250°C and 270°C, respectively. The temperature program was as follows: initial temperature 120°C for 5 min, which was increased at 3°C/min to 220°C and held at this level for 10 min (total run time: 48 min); carrier gas: He, 30 mL/min; detector gas flows: H_2_, 40 mL/min; air, 400 mL/min. Data acquisition and processing were performed with Shimadzu software for GC systems.

### 2.4. Statistical Analysis

Statistical analyses were performed with the Statistical Package for the Social Sciences (SPSS statistical software, version 21.0, IBM Corp.®, USA). The acquired data (clinical, biological, and fatty acids profiles) were divided into two different pairs: diabetic/not diabetic and inaugural diabetic/not inaugural diabetic. Clinical and biological data were reported as the mean and standard deviation (SD), as well as median and respective interquartile range (IQR), and the lipid profile data for these groups were reported as the median and respective interquartile range (IQR). The Mann–Whitney test was used to compare the median of the fatty acid percentage between the groups. All probabilities with *P* values < 0.05 were regarded as significant.

## 3. Results

### 3.1. Plasmatic Fatty Acids Profile

*Inaugural Diabetes versus Noninaugural Diabetes.* Significant differences between these two groups of children were observed. There was a significant difference in the presence of saturated fatty acids, long-chain saturated fatty acids, palmitic acid, and palmitoleic acid, with higher levels at the time of diagnosis. Moreover, we observed higher levels of omega-6 fatty acids in diabetic children (Table 2).

*Inaugural Diabetes versus Controls.* Children with a recent diagnosis of diabetes had higher levels of omega-3 fatty acids, alpha-linolenic acid, and DHA. Healthy children from the control population had a higher ratio of LA/ALA (linoleic acid/alpha-linolenic acid).

*Noninaugural Diabetes versus Controls.* The EPA/AA ratio (eicosapentaenoic acid/arachidonic acid) and the amounts of saturated fatty acids, omega-9 fatty acids,* cis*-monounsaturated fatty acids, saturated long-chain fatty acids, palmitic acid, and palmitoleic acid were higher in the control population. The diabetic children had higher levels of omega-6 fatty acids, polyunsaturated fatty acids,* cis*-pentadecenoic acid, heptadecenoic acid, linoleic acid, linolenic acid, and arachidonic acid (Table 3).

## 4. Discussion

Contrary to findings from other studies, the levels of omega-3 fatty acids were higher in the recent diabetes group relative to controls. However, other studies have found no relationship between the presence of these fatty acids and diabetes [13]. The amount of omega-6 fatty acids was actually higher in the control population. The content of fatty acids in the control population, which was made up of healthy children with a normal BMI, raises questions about the diet of these children.

We found that omega-6 levels were higher in the nonrecent diabetes group compared to the recent diabetes and to the control groups. In addition, linoleic acid levels were higher in the recent diabetes group. Kurotani et al. have already described an inverse relationship between the levels of C-peptide and this fatty acid [13].

An inhibitory effect of palmitic acid on the production and metabolic action of insulin has been described [14]. Curiously, our results showed higher levels of this fatty acid in the recent diabetes group.

Several studies have also shown a relationship between elevated levels of palmitic and palmitoleic acids and low levels of linolenic and linoleic acids with insulin resistance markers [15–17].

It is interesting to note that the omega-6/omega-3 ratio did not fall within the recommended values (3/1) in either group (inaugural diabetes: 7.9/1; noninaugural diabetes: 9.2/1; controls: 9.3/1) [18]. The polyunsaturated fatty acids were more abundant in children with established diabetes, even when compared to controls. There was an inverse relationship with respect to saturated fatty acids and long-chain fatty acids, which were lower in the children with diabetes. Although many different fatty acids have statistically significant relationships between these three groups, some seem to detach due to very low levels of alpha-linolenic acid in either the control or the inaugural diabetes group. Furthermore, curiously, EPA and DHA were more abundant in the children with diabetes compared with the control population and the inaugural diabetes group.

All of these differences were found in children with identical total triglycerides, though cholesterol levels were higher at the time diabetes was diagnosed. The overall lipid profile was not assessed in the controls (Table 4).

## 5. Conclusion

Our results showed that children with diabetes had higher levels of alpha-linolenic acid, EPA, and DHA, as well as mono- and polyunsaturated fatty acids. This observation is attributed to pharmacological therapy and nutritional management of these children. Indeed, knowing the crucial role of food in the treatment and control of type 1 diabetes is part of the follow-up of these patients, which is supposed to review and guide them from a nutritional point of view. Particular emphasis should be placed on the importance of maintaining a healthy diet, with an adequate component of fruits and vegetables as well as diversification of animal sources. Note that the fatty acid profile of diabetic children appears to be healthier than that of children in their inaugural episode as well as healthy children, leading the authors to highlight the importance of early nutritional intervention.

It is also noted that “healthy” Portuguese children show high levels of saturated fatty acids, as well as an omega-6/omega-3 ratio that is much higher than recommended. Even though we live in a country where, ideally, a so-called Mediterranean diet is practiced, the authors question whether the younger members of our society are actually consuming this type of diet.

## Figures and Tables

**Table 1:** Characteristics of participants.

	Type 1 diabetes	New onset diabetes	Controls
Number	23	6	12
M/F ratio	12/11	3/3	7/5
Age (years)	7.15 (4.0–9.7)	7.36 (3.9–9.7)	6.6 (3.6–10.3)
BMI (kg/m^2^)	17.03	16.94	16.97
Duration of disease (years)	3.29 (0.5–7.2)		
HbA1c (%)	8.17 (6.3–10.8)	9.9 (8.2–11.9)	

**Table 2:** Plasma fatty acids.

Plasma FA	NOD			Diabetes (D)			Controls (C)			NPT		
	*n*	Mean	SD	*N*	Mean	SD	*n*	Mean	SD	NOD versus D	NOD versus C	C versus D
										*P*	*P*	*P*
Sat. FA	8	35,12	3,33	34	31,77	2,87	10	34,45	1,39	0,009	0,897	0,004
Omega-3	8	4,37	1,05	34	4,21	0,98	10	3,76	0,52	0,44	0,043	0,085
Omega-6	8	34,82	4,18	34	39,11	3,85	10	34,97	2,87	0,012	0,829	0,001
LA/ALA	7	220,45	86,35	28	184,82	101,94	9	498,84	285,05	0,199	0,042	0,002

NOD: new onset diabetes; Sat. FA: saturated fatty acids; SD: standard deviation; NPT: nonparametric test.

**Table 3:** Plasma fatty acid profile.

Plasma fatty acid profile	New onset diabetes			Diabetes			Controls			NPT		
	*n*	Mean	SD	*N*	Mean	SD	*n*	Mean	SD	Nod versus D	Nod versus C	C versus D
										*P*	*P*	*P*
Butyric acid	8	1,55	2,31	34	0,78	1,18	10	1,19	0,74	0,199	0,515	0,058
Caproic acid	6	0.03	0.02	29	0.07	0.04	9	0.03	0.01	**0.014**	0.955	**0.004**
Lauric acid	7	0.14	0.11	11	0.06	0.02	9	0.10	0.07	0.056	0.408	0.056
Myristic acid	8	1.05	0.55	34	0.67	0.30	10	0.83	0.25	**0.037**	0.408	0.096
Pentadecanoic acid	8	0.28	0.12	32	0.29	0.26	10	0.29	0.14	0.415	0.762	0.358
Palmitic acid	8	23.82	2.36	34	21.01	1.71	10	22.99	1.48	0.001	0.515	**0.002**
Palmitoleic acid	8	1.71	0.80	34	1.03	0.38	10	1.75	0.44	**0.022**	0.515	0.0004
Heptadecanoic acid	8	0.29	0.07	34	0.25	0.07	10	0.24	0.05	0.114	0.068	0.710
Stearic acid	8	7.86	0.98	34	8.47	1.56	10	8.62	0.71	0.210	0.083	0.945
Oleic acid	8	22.92	2.52	34	22.35	4.87	10	24.04	1.67	0.320	0.315	0.031
Linoleic acid	8	26.82	3.55	34	30.72	3.67	10	27.87	3.00	**0.012**	0.573	**0.041**
*γ*-Linolenic acid	8	0.54	0.20	34	0.50	0.12	10	0.64	0.32	0.937	0.515	0.066
ALA	7	0.14	0.08	28	0.20	0.09	9	0.08	0.06	0.104	**0.042**	**0.0002**
Arachidonic acid	8	7.23	1.45	34	7.70	1.69	10	6.22	0.85	0.304	0.101	**0.001**
DHA	8	2.12	0.75	34	1.95	0.74	10	1.35	0.33	0.582	**0.016**	**0.002**

ALA: *α*-linolenic acid; NPT: nonparametric test.

**Table 4:** Plasma lipid profile.

Plasma lipid profile	New onset diabetes					Diabetes					Nonparametric test
	*N*	Min.	Max.	Mean	SD	*n*	Min.	Max.	Mean	SD	NOD versus D
Total cholesterol (mg/dl)	5	175.0	215.0	187.0	17.13	31	123.0	224.0	162.26	22.34	0.012
Total triglycerides (mg/dl)	5	50.0	81.0	62.8	12.28	30	31.0	118.0	59.43	20.92	0.477
HDL cholesterol (mg/dl)	5	80.0	150.0	107.8	27.22	30	52.0	135.0	91.7	19.16	0.321
LDL cholesterol (mg/dl)	5	49.0	87.0	66.6	13.59	31	21.0	75.0	58.1	11.09	0.282

## Abbreviations

BMI: Body mass index
NF-kB: Nuclear factor-kB
INF-*γ*: Interferon-*γ*
EPA: Eicosapentaenoic acid
DHA: Docosahexaenoic acid
BHT: Butylated hydroxytoluene
LA/ALA: Linoleic acid/alpha-linolenic acid
EPA/AA: Eicosapentaenoic acid/arachidonic acid.

## References

[1] Norris J. M., Kroehl M., Fingerlin T. E. (2014). Erythrocyte membrane docosapentaenoic acid levels are associated with islet autoimmunity: the Diabetes Autoimmunity Study in the Young. *Diabetologia*.

[2] Calder P. C., Grimble R. F. (2002). Polyunsaturated fatty acids, inflammation and immunity. *European Journal of Clinical Nutrition*.

[3] Fritsche K. (2006). Fatty acids as modulators of the immune response. *Annual Review of Nutrition*.

[4] Norris J. M., Yin X., Lamb M. M. (2007). Omega-3 polyunsaturated fatty acid intake and islet autoimmunity in children at increased risk for type 1 diabetes. *Journal of the American Medical Association*.

[5] Balk E. M., Lichtenstein A. H., Chung M., Kupelnick B., Chew P., Lau J. (2006). Effects of omega-3 fatty acids on serum markers of cardiovascular disease risk: a systematic review. *Atherosclerosis*.

[6] Calder P. C. (2009). Polyunsaturated fatty acids and inflammatory processes: new twists in an old tale. *Biochimie*.

[7] Serhan C. N. (2010). Novel lipid mediators and resolution mechanisms in acute inflammation: to resolve or not?. *American Journal of Pathology*.

[8] Harris S. G., Padilla J., Koumas L., Ray D., Phipps R. P. (2002). Prostaglandins as modulators of immunity. *Trends in Immunology*.

[9] Luo P., Wang M. H. (2011). Beta-cell function and diabetes. *Prostaglandins & Other Lipid Mediators*.

[10] Hua Q., Liang J., Chen, Wang Y. (2011). Development of a derivatization-free GC-FID method for evaluation of free fatty acid levels in plasma of diabetic nephropathy patients. *Chemical Research in Chinese Universities*.

[11] Seppänen-Laakso T., Laakso I., Hiltunen R. (2002). Analysis of fatty acids by gas chromatography, and its relevance to research on health and nutrition. *Analytica Chimica Acta*.

[12] Bondia-Pons I., Moltó-Puigmartí C., Castellote A. I., López-Sabater M. C. (2007). Determination of conjugated linoleic acid in human plasma by fast gas chromatography. *Journal of Chromatography A*.

[13] Kurotani K., Sato M., Ejima Y. (2012). High levels of stearic acid, palmitoleic acid, and dihomo-*γ*-linolenic acid and low levels of linoleic acid in serum cholesterol ester are associated with high insulin resistance. *Nutrition Research*.

[14] Zahradka P., Wigle J., Pierce G. N. (2003). High levels of palmitic acid lead to insulin resistance due to changes in the level of phosphorylation of the insulin receptor and insulin receptor-1. *Molecular and Cellular Biochemistry*.

[15] Wang L., Folsom T., Zheng Z., Pankow J., Eckfeldt J. (2003). Plasma fatty acid composition and incidence of diabetes in middle-aged adults: the Atherosclerosis Risk in Communities (ARIC) Study. *The American Journal of Clinical Nutrition*.

[16] Gustavo Vazquez-Jimenez J., Chavez-Reyes J., Romero-Garcia T. (2016). Palmitic acid but not palmitoleic acid induces insulin resistance in a human endothelial cell line by decreasing SERCA pump expression. *Cellular Signalling*.

[17] Bei F., Jia J., Jia Y.-Q. (2015). Long-term effect of early postnatal overnutrition on insulin resistance and serum fatty acid profiles in male rats. *Lipids in Health and Disease*.

[18] Perini J. Â. D. L., Stevanato F. B., Sargi S. C. (2010). Omega-3 and omega-6 polyunsaturated fatty acids: metabolism in mammals and immune response. *Revista de Nutricao*.

